# Rehabilitation after surgical release of the stiff elbow: A literature review^☆^

**DOI:** 10.1016/j.jor.2024.10.061

**Published:** 2024-11-10

**Authors:** M.M. Schneider, V. Rentschler, S. Geyer, C. Jung, B. Hollinger, F. Pfalzer, K. Beitzel, K. Burkhart, C. Schoch

**Affiliations:** a Praxisklinik Orthopädie Aachen (PKO), Germany; b University of Witten / Herdecke, Germany; c Sektion für Gelenk- und Extremitätenchirurgie, Uniklinik RWTH Aachen, Germany; d Klinik für Unfall-, Handchirurgie und Sportmedizin, ViDia Kliniken, Karlsruhe, Germany; e St. Vinzenz Klinik Pfronten, Pfronten, Germany; f Orthopädie Ost, Will, Switzerland; g Zentrum für Sportorthopädie und Gelenkchirurgie, Orthopädische Klinik Markgröningen, Markgröningen, Germany; h Sportpraxis Stuttgart, Stuttgart, Germany; i Schulterinstitut Beitzel, ATOS Orthoparc Klinik, Cologne, Germany; j Diakonie Klinikum, Stuttgart, Germany

**Keywords:** Osteocapsular release, Elbow stiffness, Rehabilitation, Aftercare, Arthrolysis, Debridement arthroplasty

## Abstract

**Background:**

Elbow stiffness poses a significant challenge for surgeons as well as physiotherapists during and after surgery. To date, there is no consensus regarding the subsequent rehabilitation after surgical release of the stiff elbow.

**Objective:**

The aim is to evaluate the most important therapeutic strategies following open or arthroscopic release of the stiff elbow based on a comprehensive literature review, and to develop a consensus for or against specific therapeutic methods with the help of a survey among elbow experts of the D-A-CH Association for Shoulder and Elbow Surgery (DVSE).

**Methods:**

Literature search was performed based on guidelines, the “health technology assessments”, systematic reviews and clinical studies that examined rehabilitation after osteocapsular release of the stiff elbow. The databases of the “Guidelines International Network”, various national guidelines, PubMed, the “Cochrane Central Register of Controlled Trials”, the “Cochrane Database of Systematic Reviews”, and the “Physiotherapy Evidence Database” were scanned, each for the period from January 1989 to December 2019. As part of an online survey, all active members of the DVSE were asked about their strategies in immediate aftercare and rehabilitation after elbow arthrolysis.

**Results:**

A total of 5 reviews and 55 articles could be identified from 107 articles since 1989, which served as the basis for the preparation of an evidence-based aftercare recommendation. By reviewing all the mentioned paper and evaluation of the survery of DVSE members, a basic concept could be finalized.

## 1 Introduction

Restrictions in elbow range of motion can have various causes, which were described as intrinsic, extrinsic or mixed.^1^ Elbow stiffness can occur following trauma, surgical procedures or in the context of degenerative diseases.^2^ Typically, the development of elbow stiffness involves a combination of both intrinsic and extrinsic causes.^3^ The elbow joint is prone for the development of arthrofibrosis and elbow stiffness due to various aspects. The causes include the high congruence of the joint, the appearance of the synovium and the stabilizing function of the joint capsule and ligaments.

Contractures and inflammatory processes occur quicker and more frequent even in minor injuries or short-term immobilization compared to other joints.^4^ The joint capsule tends to shrink, especially in prolonged immobilization and in the setting of heterotopic ossifications resulting in varying degrees of elbow stiffness.^5^

Pro-inflammatory cytokines and interleukins are released during the formation of soft tissue contractures and inflammatory processes. These lead, among other things, to an enhanced transformation of fibroblasts into myofibroblasts^6^ and, on the other hand, to an increased production of the growth factor TGF-β.^7^, ^8^, ^9^ As a result, arthrofibrotic tissue forms, which is characterized by an increased formation of collagen cross-links and a decreased proteoglycan, lubricin, and water content.^6^^,^^9^^,^^10^ This process is amplified by the lack of mechanical stress during immobilization.^5^

Capsular contracture with elbow stiffness can lead to significant limitations in today's daily life. As early as 1996, Søjbjerg et al. indicated that a 50 % reduction in elbow range of motion could result in a functional loss of the upper extremity of up to 80 %.^11^ A physiological range of motion of the elbow joint is essential for the unrestricted execution of many activities of daily living (ADL). The functional 'arc of motion' of 100° for both extension/flexion and pro-/supination, described by Morrey, seems no longer appropriate.^1^

In our current times, the requirements for the elbow joint have changed. With increasing tasks involving the use of keyboards and computer mice and the widespread adoption of mobile phones, there is a need for a greater range of motion to fulfill all ADL.^12^, ^13^, ^14^, ^15^

Restrictions in range of motion of the elbow joint following dislocation, fracture, or a combination of both range from 1.2 % to 12 %.^16^^,^^17^ Elbow stiffness caused by degenerative changes seem to be less common with a prevalence of 2 %.^18^ Furthermore, elbow contracture can occur after infections, burns and proliferative diseases such as chondromatosis.^19^, ^20^, ^21^, ^22^ Elbow stiffness can be classified according to Morrey and Kay.^23^^,^^24^

While Kay's classification differentiates between five distinct structural changes in elbow stiffness,^23^ Morrey's classification, on the other hand, is based on etiological and anatomical aspects.^24^ The decision for surgical release of the stiff elbow depends on the degree of restriction in range of motion (minimal: up to 90°, moderate: 61°–90°, severe: 31°–60°, and very severe: 30°), the type of stiffness (flexion contracture vs. extension limitation or mixed forms), the presence of implants or the existence of heterotopic ossifications, as well as neurological pathologies (ulnar nerve entrapment).^18^^,^^25^, ^26^, ^27^ When non-surgical measures do not lead to sufficient improvement, surgery offers a viable option with a high rate of patient satisfaction. Over time, the arthroscopic approach for the release of the stiff elbow has become increasingly popular and is recognized as a safe and efficient procedure.^28^, ^29^, ^30^, ^31^, ^32^ Despite considerable improvements in the surgical techniques and distinct treatment concepts, there is substantial variance in the literature regarding immediate and medium-term aftercare. Rehabilitation protocols and postoperative therapy recommendations range from self-exercises with stretching^33^ to 'continuous passive motion' (CPM) therapy^34^^,^^35^ up to the use of extension splints and treatment with external fixators.^36^

The use of peripheral analgesia methods such as a catheter anesthesia is as controversially discussed as the administration of oral medications for the prevention of heterotopic ossification.^37^^,^^38^ Up to date, evidence-based recommendations, or even a gold standard, do not exist. The variability in postoperative strategies derive largely from a lack of conclusive data supporting one approach over another. With many rehabilitation protocols grounded in tradition rather than empirical evidence, it becomes crucial to evaluate their efficacy critically. The Rehabilitation Commission of the DACH Society for Shoulder and Elbow Surgery (DVSE) and the Elbow Commission of the Society for Arthroscopy and Joint-Surgery (AGA) attempted to address the issue. The objective was to bridge the gap in understanding, ensuring that postoperative recommendations are both evidence-based and feasible for patients, physiotherapists, and surgeons. The present study aimed to evaluate various postoperative treatment options after surgical release of the stiff elbow in an evidence-based manner based on a comprehensive literature review. Additionally, a survey conducted among elbow specialists from the DVSE, was thought to determine the prevailing consensus regarding specific rehabilitation approaches. We aim to facilitate the establishment of a guideline for aftercare, aligning with the principles of "best clinical practice."

## 2 Methods

### 2.1 Review of the literature

**Fig. 1:**
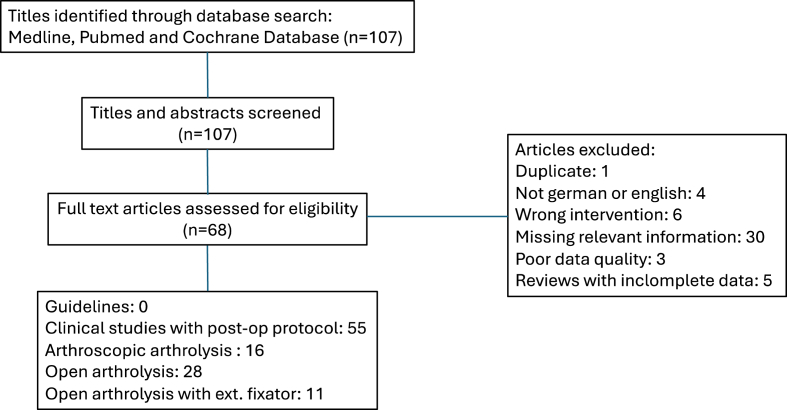
Preferred Reporting Items for Systematic Reviews and Meta-analyses (PRISMA) flow diagram demonstrating systematic review of literature for surgical release of the stiff elbow.

The systematic literature search followed the standard guideline for systematic reviews and meta-analyses (PRISMA).^39^ Studies that investigated the postoperative management after surgical release of elbow contracture with evidence levels between grades I-IV, guidelines and case reports in English and German were considered suitable. We excluded expert opinions without evidence as well as review articles and "letters to the editor”. The authors conducted a review with the help of the following medical databases: PubMed, Cochrane Central Register of Controlled Trials und Cochrane Database of Systematic Reviews. The search was conducted on June 12th^,^ 2019. The search terms used were "elbow stiffness," "elbow arthrolysis," "elbow rehabilitation," and "elbow release". We included studies published between 1989 and 2019. The results were checked for duplicates and matching our inclusion criteria. We identified five reviews, none of which gave distinct or evidence-based recommendations on how to treat patients after surgery for elbow stiffness. As mentioned before, reviews were excluded. An overview of our conducted literature review is shown in Fig. 1.

The articles evaluated in the final analysis were sorted and evaluated using the "Level of Evidence"^40^ and the so-called PICO concept.^41^, ^42^, ^43^

The assessment was conducted by six investigators and all articles were reviewed based on the following criteria: study design, demographic data of the study population, average follow-up period after surgery, type of surgery (arthroscopic vs. open), surgical setting of the intervention (outpatient vs. inpatient), pre- and postoperative range of motion, postoperative pain management, postoperative rehabilitation, complications, and clinical outcomes. The data was analyzed using Microsoft Excel (Version 16.55, Microsoft, Redmond, Washington, USA).

### 2.2 Online survey of experts

The elaboration of the expert opinion was conducted as an online survey using an online platform (www.surveymonkey.com) and aimed to capture the "best clinical practice." After reviewing the literature, the Rehabilitation Comittee of the DACH Society for Shoulder and Elbow Surgery (DVSE) developed specific questions regarding the surgical elbow arthrolysis and its postoperative management, which are detailed in Table 1, Table 2, Table 3 along with the given answers.

**Table 1:**
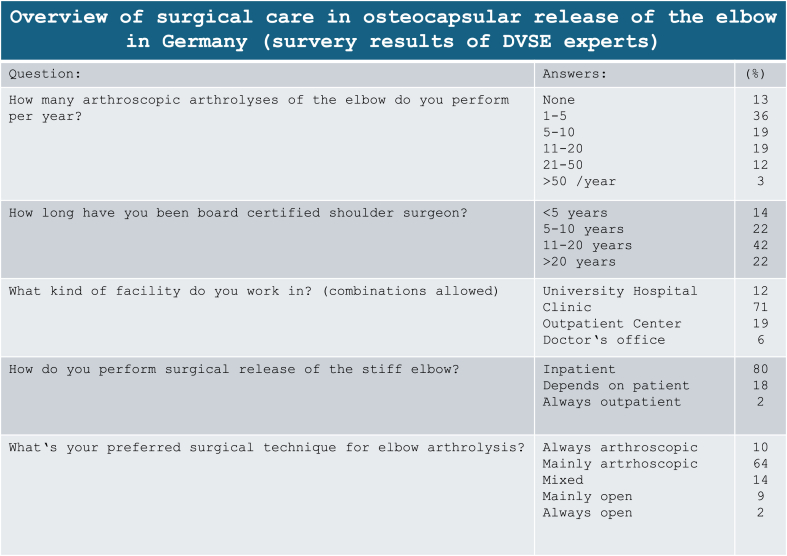
Experience and favorite approach of the participants of the survey.

**Table 2:**
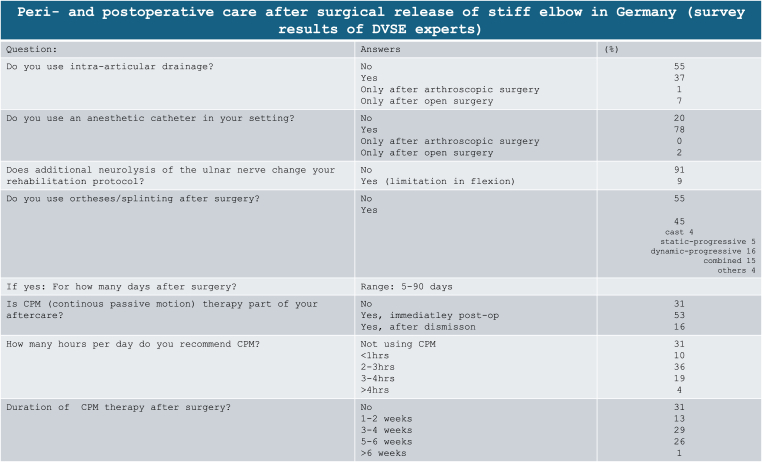
Survey results on peri- and postoperative management after surgical release of stiff elbow.

**Table 3:**
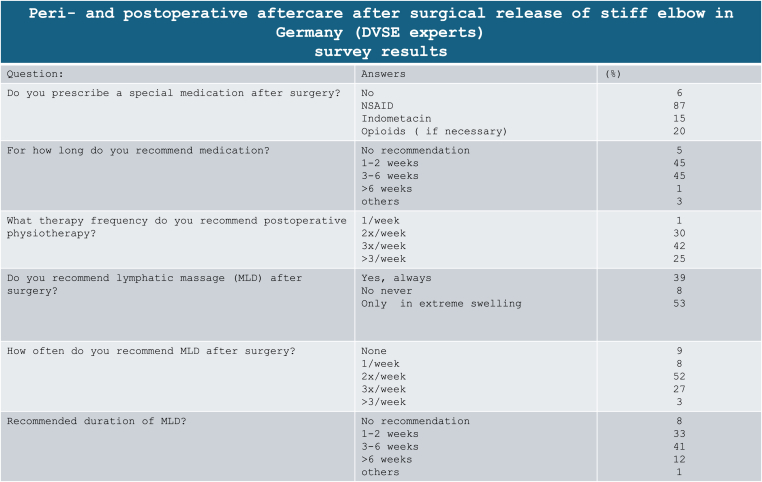
Postoperative management concerning pain killers, physiotherapy and lymphatic drainage.

The online survey was sent to members of the DVSE. Participants were provided a link to an online questionnaire consisting of 19 questions. The survey was sent to a total of over 700 members. Experts were reminded to participate in the survey thrice, which was conducted from October 1, 2019, to November 30, 2019. Physicians who do not perform elbow arthrolysis were excluded from the survey. The statistical analysis of the responses was carried out automatically using SurveyMonkey and Microsoft Excel (Version 16.55, Microsoft, Redmond, Washington, USA).

## 3 Results and discussion

While conducting the systematic literature search, we were unable to identify any guidelines or even a gold standard for postoperative care following arthrolysis in both the Anglo-American and German literature. Out of the 266 articles reviewed, 63 met the inclusion criteria and were subjected to evaluation. Among these 63 articles, 5 were systematic reviews, and 58 were clinical studies.

In total, the survey recorded 112 participants, with 97 surveys being completed in their entirety. 86 % of the respondents indicated having more than five years of experience in elbow surgery, and 64 % even reported having over ten years of experience.

In the following, we present and discuss the results of the literature review while incorporating the expert opinions from the survey.

### 3.1 Surgical release of the stiff elbow

The surgical release of the stiff elbow has become an established and effective option, as conservative treatment often fails to achieve sufficient improvement.^23^^,^^44^^,^^45^

In cases of unsuccessful conservative treatment, both arthroscopic and open surgery can reduce pain and improve elbow range of motion with a high likelihood of success, regardless of the underlying cause of stiffness.^28^^,^^29^^,^^31^^,^^46^^,^^47^ There is no consensus in the literature regarding the optimal surgical technique for elbow stiffness..^18^^,^^48^, ^49^, ^50^

### 3.2 Arthroscopic versus open surgery

The evaluation of the expert survey revealed that 74 % of the respondents mostly perform arthroscopic arthrolyses, 11 % primarily performed open surgery, while the remaining 14 % employed a combination of both arthroscopic and open approaches (Table 1).

As anticipated, studies focusing on arthroscopic arthrolysis have mainly emerged within the past decade. 71 % of survey participants conducted their surgical procedures in the setting of a hospital, with 90 % of all procedures being scheduled as inpatient surgeries. The literature review revealed an average in-patient stay ranging from 3 to 21 days in 15 out of 16 studies of patients undergoing open surgery. Similarly, arthroscopic surgery usually was performed as in-patient procedure while the option of an outpatient care was not mentioned in any studies.

### 3.3 Postoperative drainage

Most DVSE experts did not use drains after surgery (56 %). 37 % indicated that the decision to place a drain or not was made during surgery, while only 7 % routinely used drains after an open approach. This is backed up by the literature: Without stating any reasons, Olivier et al. as well as Lim et al. used drains in all cases after open arthrolysis for fracture sequelae (n = 91) and arthroscopic procedures (n = 43), respectively.^51^^,^^52^ On the other hand, Mansat et al. describe that the need for postoperative drainage depends on the complexity of the procedure and the severity of elbow stiffness. Accordingly, in complex cases with open surgical release, drainage should always be used to prevent postoperative hematomas, which can affect outcomes and complications.^53^ However, in most studies, postoperative drains are not mentioned, making it difficult to draw clear evidence-based conclusions.

### 3.4 Postoperative pain management

Regional analgesia (RA) is considered the preferred method of pain management in both open and arthroscopic surgery and is favored over systematic pharmacotherapy.^54^ This aligns with the statements of the experts in our survey, with 80 % resorting to RA using a catheter for postoperative pain management. In the literature, the use of a catheter in arthroscopic release of the stiff elbow was only mentioned in five out of 16 studies.^31^^,^^47^^,^^52^^,^^55^^,^^56^ In open surgery, the use of RA was mentioned in 12 studies, and it was regularly employed in ten of them.^33^^,^^51^^,^^53^^,^^55^, ^56^, ^57^, ^58^, ^59^, ^60^, ^61^, ^62^, ^63^, ^64^

Only the studies by Lahoda et al. (12 days) and Pettersen et al. (9 days) stated the duration the pain catheter was used.^60^^,^^61^

The duration the catheter remained after surgery was not queried in the survey. Literature does not provide details concerning oral pharmacotherapy after surgery for elbow stiffness. In most studies, there is no statement regarding postoperative pain management. In the expert survey, 86.6 % of respondents indicated that they administer non-steroidal anti-inflammatory drugs (NSAIDs) and, if needed, additional opioid analgesics after surgery. Among them, 45 % selected a duration of 1–2 weeks, while another 45 % opted for 3–6 weeks. Heterotopic ossification after surgical release of the elbow is rare but feared complication. Therefore, a prophylaxis for heterotopic ossification can be initiated immediately after surgery. The literature has no consensus concerning pharmacotherapy for the prophylaxis of heterotopic ossification. In 16 studies, oral medication with indomethacin (varying duration from a single dose preoperatively to continued intake for 6 weeks after surgery) was routinely performed.^46^^,^^56^^,^^58^^,^^60^^,^^62^^,^^63^^,^^65^, ^66^, ^67^, ^68^, ^69^, ^70^, ^71^, ^72^, ^73^, ^74^, ^75^ The most frequently described duration was three weeks. Rai et al., on the other hand, used Naproxen for a total of two months instead.^30^ Sun et al. And three other groups prescreibed Celecoxib 200 mg twice a day for seeks weeks.^76^, ^77^, ^78^, ^79^

The remaining studies provide no statement regarding prophylaxis for heterotopic ossificiation. In the survey, only 15 % of respondents used Indomethacin. Currently, there is no recommendation for oral medication for optimal prophylaxis of heterotopic ossification following fractures or surgeries.^80^, ^81^, ^82^ Some studies even doubt the additional benefit of NSAIDs for reducing the risk of heterotopic ossification after elbow arthrolysis.^83^

### 3.5 Physical therapy after surgery

There is also a lack of consensus in terms of physical therapy, the use of various braces and continuous passive motion (CPM) devices. CPM therapy is mentioned in eleven out of 16 studies after arthroscopic surgery. However, only some studies provide details regarding frequency and duration. While Cefo et al.^84^ and Achtnich et al.^55^ limited CPM therapy to 24 h postoperatively or the inpatient stay, Rai et al.^30^ and Willinger et al.^31^ conducted the use of CPM devices for 3 × 30 min per day without stating a time frame and 8 × 20 min daily for 3 weeks, respectively.

In the 28 studies on open arthrolysis involving 1245 patients, the use of CPM devices is described in 23 articles.^30^^,^^33^^,^^46^^,^^53^^,^^56^^,^^57^^,^^59^^,^^60^^,^^72^, ^73^, ^74^, ^75^^,^^85^, ^86^, ^87^, ^88^, ^89^, ^90^, ^91^, ^92^, ^93^, ^94^, ^95^ While two papers completely abstained from continuous passive motion therapy,^69^^,^^94^ the remaining studies did not mention it. Details regarding duration and frequency were often not provided. When mentioned, the time frame of CPM therapy varied from one day^51^ to as long as 42 days^61^ and was used in one study for up to 12 h per day.^57^ According to our survey, 52.6 % of the participants provide a CPM machine during the patient's stay in the hospital. However, only 16 % of the experts continued using motion therapy after discharge. Concerning frequency, 36 % utilized it for 2–3 h, 18 % for 3–4 h, and 4 % for more than 4 h per day. 13 % of doctors deploying CPM therapy recommended it for 1–2 weeks, 29 % for 3–4 weeks, and 25 % for 5–6 weeks after surgery(see Table 2)

Physiotherapy is considered a standard following arthroscopic arthrolysis. Experts of the DVSE recommended it with a frequency of 2 times per week in 30 %, 3 times per week in 42 %, and more than 3 times per week in 25 %.

In the literature, nearly all studies consistently address postoperativ physical therapy. However, most papers typically provide limited details about the type and extent of physiotherapy. In two studies from Germany postoperative physiotherapy was performed for 6 weeks.^31^^,^^55^ Lubiatowski et al. and Ayadi et al. extended the duration to 6 and 9 months, respectively.^56^^,^^96^ In two studies, physical therapy was explicitly not initiated.^46^^,^^59^ The need for and the effect of lymphatic drainage remains unclear, since it is infrequently mentioned in the literature and its prescription varies. Only 39 % of survey participants routinely initiated it after surgery, while 53 % indicated that they consider lymphatic drainage based on postoperative elbow and forearm swelling. In doctors prescribing lymphatic drainage, 33 % recommended it for 1–2 weeks, 41 % for 3–4 weeks, and 13 % for more than 5 weeks (Table 3).

### 3.6 Use of postoperative casts and splints

The use of temporary extension or flexion splints is discussed in numerous studies (12 times after open and nine times after arthroscopic release of the stiff elbow).^30^^,^^31^^,^^51^, ^52^, ^53^^,^^57^^,^^63^^,^^68^, ^69^, ^70^^,^^72^^,^^74^^,^^75^^,^^86^, ^87^, ^88^^,^^92^, ^93^, ^94^^,^^97^, ^98^, ^99^, ^100^, ^101^, ^102^, ^103^, ^104^, ^105^, ^106^, ^107^ For example, Tan et al. applied an extension splint for 24 h after open arthrolysis.^75^ In an earlier study by Hertel and colleagues, a splint was recommended for 8 weeks postoperatively. During the day, an hourly switch between extension and flexion splinting was performed, and extension positioning was maintained during the night.^90^ Nowadays, maintaining such compliance seems challenging. There is no clear recommendation for postoperative casting or treatment with orthesis; instead, preference and experience of the surgeon play a crucial role. In the expert survey, 55 % stated that they do not perform any splinting at all after surgery. However, 17 % confirmed the regular use of dynamically progressive splints over a period of 2–42 days, sometimes only at night.

## 4 Conclusion

In summary, there is no gold standard for postoperative management following surgical release of the stiff elbow. Our survey offered variable approaches regarding pain management and postoperative care. No significant difference in postoperative management between open and arthroscopic surgery was identified. Elbow releases are predominantly performed in an inpatient setting. In most cases, postoperative pain management involves the use of regional catheters and additional NSAIDs. Based on the survey or literature review, a recommendation for oral medication to prevent heterotopic ossification cannot be made. Physiotherapy is prescribed in almost all cases to maintain the regained range of motion, with the duration of therapy varying and likely requiring an individual approach for each patient. The use of splints does not impact postoperative outcomes significantly and is applied rarely, most of the times based on the surgeon's preference. The use of CPM machines is mentioned in most studies, although the duration and intensity vary significantly.

## Guardian/Patient's consent

Not applicable, systematic review.

## Author contributions

Concept B.H., F.P., C.S. and C.J.; methodology C.S., C.J., K.B.; Data curation V.R., S.G.; Writing and draft preparation M.M.S., C.S., V.R., K.J.B., K.B., C.J. and S.G.; Review and Editing, M.M.S, C.S.; Supervision, C.S.; Project administration: M.M.S., C.S.; Admission: M.M.S.

## Ethical statement

Systematic review, not applicable.

## Funding

This research received no specific grant from any funding agency in the public, commercial, or not-for-profit sectors.

## Conflict of interest

Neither author, their immediate family, and any research foundation with which they are affiliated did not receive any financial payments or other benefits from any commercial entity related to the subject of this article.

## References

[1] Morrey B.F., Askew L.J., Chao E.Y. (1981). A biomechanical study of normal functional elbow motion. J Bone Joint Surg Am.

[2] Attum B., Obremskey W. (2016). Posttraumatic elbow stiffness: a critical analysis review. JBJS Rev.

[3] Zhang D., Nazarian A., Rodriguez E.K. (2020). Post-traumatic elbow stiffness: pathogenesis and current treatments. Shoulder Elbow.

[4] Mattyasovszky S.G., Hofmann A., Brochhausen C. (2010). The effect of the pro-inflammatory cytokine tumor necrosis factor-alpha on human joint capsule myofibroblasts. Arthritis Res Ther.

[5] Akeson W.H., Amiel D., Mechanic G.L., Woo S.L., Harwood F.L., Hamer M.L. (1977). Collagen cross-linking alterations in joint contractures: changes in the reducible cross-links in periarticular connective tissue collagen after nine weeks of immobilization. Connect Tissue Res.

[6] Unterhauser F.N., Bosch U., Zeichen J., Weiler A. (2004). Alpha-smooth muscle actin containing contractile fibroblastic cells in human knee arthrofibrosis tissue. Winner of the AGA-DonJoy Award 2003. Arch Orthop Trauma Surg.

[7] Bunker T.D., Reilly J., Baird K.S., Hamblen D.L. (2000). Expression of growth factors, cytokines and matrix metalloproteinases in frozen shoulder. J Bone Joint Surg Br.

[8] Younai S., Venters G., Vu S., Nichter L., Nimni M.E., Tuan T.L. (1996). Role of growth factors in scar contraction: an in vitro analysis. Ann Plast Surg.

[9] Watson R.S., Gouze E., Levings P.P. (2010). Gene delivery of TGF-beta1 induces arthrofibrosis and chondrometaplasia of synovium in vivo. Lab Invest.

[10] Lubis A.M., Lubis V.K. (2013). Matrix metalloproteinase, tissue inhibitor of metalloproteinase and transforming growth factor-beta 1 in frozen shoulder, and their changes as response to intensive stretching and supervised neglect exercise. J Orthop Sci.

[11] Søjbjerg J.O. (1996). The stiff elbow. Acta Orthop Scand.

[12] Sardelli M., Tashjian R.Z., MacWilliams B.A. (2011). Functional elbow range of motion for contemporary tasks. J Bone Joint Surg Am.

[13] Oosterwijk A.M., Nieuwenhuis M.K., van der Schans C.P., Mouton L.J. (2018). Shoulder and elbow range of motion for the performance of activities of daily living: a systematic review. Physiother Theory Pract.

[14] Valone L.C., Waites C., Tartarilla A.B. (2020). Functional elbow range of motion in children and adolescents. J Pediatr Orthop.

[15] Raiss P., Rettig O., Wolf S., Loew M., Kasten P. (2007). [Range of motion of shoulder and elbow in activities of daily life in 3D motion analysis]. Z für Orthop Unfallchirurgie.

[16] Modi C.S., Wasserstein D., Mayne I.P., Henry P.D., Mahomed N., Veillette C.J. (2015). The frequency and risk factors for subsequent surgery after a simple elbow dislocation. Injury.

[17] Myden C., Hildebrand K. (2011). Elbow joint contracture after traumatic injury. J Shoulder Elbow Surg.

[18] Kodde I.F., van Rijn J., van den Bekerom M.P., Eygendaal D. (2013). Surgical treatment of post-traumatic elbow stiffness: a systematic review. J Shoulder Elbow Surg.

[19] Lee E.K., Namdari S., Hosalkar H.S., Keenan M.A., Baldwin K.D. (2013). Clinical results of the excision of heterotopic bone around the elbow: a systematic review. J Shoulder Elbow Surg.

[20] Gaur A., Sinclair M., Caruso E., Peretti G., Zaleske D. (2003). Heterotopic ossification around the elbow following burns in children: results after excision. J Bone Joint Surg Am.

[21] Kaziz H., Triki M.A., Mouelhi T., Bouattour K., Naouar N., Ben Ayeche M.L. (2019). Septic elbow arthritis in children: epidemiology and outcome. Arch Pediatr.

[22] Griesser M.J., Harris J.D., Likes R.L., Jones G.L. (2011). Synovial chondromatosis of the elbow causing a mechanical block to range of motion: a case report and review of the literature. Am J Orthoped.

[23] Jupiter J.B., O'Driscoll S.W., Cohen M.S. (2003). The assessment and management of the stiff elbow. Instr Course Lect.

[24] Morrey B.F. (1990). Post-traumatic contracture of the elbow. Operative treatment, including distraction arthroplasty. J Bone Joint Surg Am..

[25] Rausch V., von Glinski A., Königshausen M., Schildhauer T.A., Seybold D., Geßmann J. (2017). Steifer posttraumatischer ellenbogen. Trauma Berufskrankh.

[26] Leschinger T., Müller L.P., Hackl M., Wegmann M. (2016). Techniken der Arthrolyse am Ellenbogen. Obere Extremität.

[27] Willinger L., Lacheta L., Imhoff A.B., Siebenlist S. (2019). Der steife Ellenbogen (Teil 1) - Arthroskopische Arthrolyse und deren Grenzen. Arthroskopie.

[28] Sochacki K.R., Jack R.A., Hirase T. (2017). Arthroscopic debridement for primary degenerative osteoarthritis of the elbow leads to significant improvement in range of motion and clinical outcomes: a systematic review. Arthroscopy.

[29] Beck C.M., Gluck M.J., Zhang Y. (2021). Outcomes of arthroscopic elbow contracture release: improvement for severe prosupination and flexion contracture. Arthroscopy.

[30] Rai S., Zhang Q., Tamang N., Jin S., Wang H., Meng C. (2019). Arthroscopic arthrolysis of posttraumatic and non-traumatic elbow stiffness offers comparable clinical outcomes. BMC Muscoskel Disord.

[31] Willinger L., Siebenlist S., Lenich A., Liska F., Imhoff A.B., Achtnich A. (2018). Arthroscopic arthrolysis provides good clinical outcome in post-traumatic and degenerative elbow stiffness. Knee Surg Sports Traumatol Arthrosc.

[32] Wu X., Wang H., Meng C. (2015). Outcomes of arthroscopic arthrolysis for the post-traumatic elbow stiffness. Knee Surg Sports Traumatol Arthrosc.

[33] Higgs Z.C., Danks B.A., Sibinski M., Rymaszewski L.A. (2012). Outcomes of open arthrolysis of the elbow without post-operative passive stretching. J Bone Joint Surg Br.

[34] Viveen J., Doornberg J.N., Kodde I.F. (2017). Continuous passive motion and physical therapy (CPM) versus physical therapy (PT) versus delayed physical therapy (DPT) after surgical release for elbow contractures; a study protocol for a prospective randomized controlled trial. BMC Muscoskel Disord.

[35] Lindenhovius A.L., van de Luijtgaarden K., Ring D., Jupiter J. (2009). Open elbow contracture release: postoperative management with and without continuous passive motion. J Hand Surg Am.

[36] Pennig D., Heck S., Mader K. (2011). [Distraction arthroplasty for treatment of posttraumatic elbow stiffness]. Orthopä.

[37] Agarwal S., Loder S., Levi B. (2017). Heterotopic ossification following upper extremity injury. Hand Clin.

[38] Sun Y., Cai J., Li F., Liu S., Ruan H., Fan C. (2015). The efficacy of celecoxib in preventing heterotopic ossification recurrence after open arthrolysis for post-traumatic elbow stiffness in adults. J Shoulder Elbow Surg.

[39] Moher D., Liberati A., Tetzlaff J., Altman D.G., The P.G. (2009). Preferred reporting Items for systematic reviews and meta-analyses: the PRISMA statement. PLoS Med.

[40] Howick J., Chalmers I., Glasziou P. (2011). https://www.cebm.ox.ac.uk/resources/levels-of-evidence/ocebm-levels-of-evidence.

[41] Oxman A.D., Sackett D.L., Guyatt G.H. (1993). Users' guides to the medical literature. I. How to get started. The Evidence-Based Medicine Working Group. Jama..

[42] Richardson W.S., Wilson M.C., Nishikawa J., Hayward R.S. (1995). The well-built clinical question: a key to evidence-based decisions. ACP J Club.

[43] Schardt C., Adams M.B., Owens T., Keitz S., Fontelo P. (2007). Utilization of the PICO framework to improve searching PubMed for clinical questions. BMC Med Inf Decis Making.

[44] Vardakas D.G., Varitimidis S.E., Goebel F., Vogt M.T., Sotereanos D.G. (2002). Evaluating and treating the stiff elbow. Hand Clin.

[45] Page C., Backus S.I., Lenhoff M.W. (2003). Electromyographic activity in stiff and normal elbows during elbow flexion and extension. J Hand Ther.

[46] Nguyen D., Proper S.I., MacDermid J.C., King G.J., Faber K.J. (2006). Functional outcomes of arthroscopic capsular release of the elbow. Arthroscopy.

[47] Pederzini L.A., Nicoletta F., Tosi M., Prandini M., Tripoli E., Cossio A. (2014). Elbow arthroscopy in stiff elbow. Knee Surg Sports Traumatol Arthrosc.

[48] Masci G., Cazzato G., Milano G. (2020). The stiff elbow: current concepts. Orthop Rev.

[49] Nandi S., Maschke S., Evans P.J., Lawton J.N. (2009). The stiff elbow. Hand (N Y).

[50] Leschinger T., Hackl M., Lenz M., Rausch V., Müller L.P., Wegmann K. (2019). A prospective comparison of short-term results after arthroscopic and open elbow procedures in elbow stiffness. Obere Extremität.

[51] Olivier L.C., Assenmacher S., Setareh E., Schmit-Neuerburg K.P. (2000). Grading of functional results of elbow joint arthrolysis after fracture treatment. Arch Orthop Trauma Surg.

[52] Lim T.K., Koh K.H., Lee H.I., Shim J.W., Park M.J. (2014). Arthroscopic débridement for primary osteoarthritis of the elbow: analysis of preoperative factors affecting outcome. J Shoulder Elbow Surg.

[53] Mansat P., Bonnevialle N., Werner B. (2011). [Indications and technique of combined medial and lateral column procedures in severe extrinsic elbow contractures]. Orthopä.

[54] (2009). Deutsche Interdisziplinäre Vereinigung für Schmerztherapie (DIVS) e.V..

[55] Achtnich A., Forkel P., Metzlaff S., Petersen W. (2013). [Arthroscopic arthrolysis of the elbow joint]. Operat Orthop Traumatol.

[56] Lubiatowski P., Ślęzak M., Wałecka J., Bręborowicz M., Romanowski L. (2018). Prospective outcome assessment of arthroscopic arthrolysis for traumatic and degenerative elbow contracture. J Shoulder Elbow Surg.

[57] Cikes A., Jolles B.M., Farron A. (2006). Open elbow arthrolysis for posttraumatic elbow stiffness. J Orthop Trauma.

[58] Kulkarni G.S., Kulkarni V.S., Shyam A.K., Kulkarni R.M., Kulkarni M.G., Nayak P. (2010). Management of severe extra-articular contracture of the elbow by open arthrolysis and a monolateral hinged external fixator. J Bone Joint Surg Br.

[59] Sharma S., Rymaszewski L.A. (2007). Open arthrolysis for post-traumatic stiffness of the elbow: results are durable over the medium term. J Bone Joint Surg Br.

[60] Lahoda L.U., Klapperich T., Hahn M.P., Muhr G. (1999). [Results of posttraumatic elbow arthrolyses: a prospective study]. Chirurg.

[61] Pettersen P.M., Eriksson J., Bratberg H. (2016). Increased ROM and high patient satisfaction after open arthrolysis: a follow-up-study of 43 patients with posttraumatic stiff elbows. BMC Muscoskel Disord.

[62] Liu S., Fan C.Y., Ruan H.J., Li F.F., Tian J. (2011). Combination of arthrolysis by lateral and medial approaches and hinged external fixation in the treatment of stiff elbow. J Trauma.

[63] Ruan H.J., Liu S., Fan C.Y., Liu J.J. (2013). Open arthrolysis and hinged external fixation for posttraumatic ankylosed elbows. Arch Orthop Trauma Surg.

[64] Parikh R.K., Rymaszewski L.R., Scott N.B. (1995). Prolonged postoperative analgesia for arthrolysis of the elbow joint. Br J Anaesth.

[65] Wang W., Zhan Y.L., Yu S.Y., Zheng X.Y., Liu S., Fan C.Y. (2016). Open arthrolysis with pie-crusting release of the triceps tendon for treating post-traumatic contracture of the elbow. J Shoulder Elbow Surg.

[66] Wang W., Liu S., Jiang S.C., Ruan H.J., He N., Fan C.Y. (2015). Limited medial and lateral approaches to treat stiff elbows. Orthopedics.

[67] Liu S., Liu J.J., Li X.J., Ruan H.J., Fan C.Y. (2013). Open arthrolysis and prosthetic replacement of the radial head for elbow stiffness associated with rotation limitation. J Shoulder Elbow Surg.

[68] Ouyang Y., Wang Y., Li F., Fan C. (2012). Open release and a hinged external fixator for the treatment of elbow stiffness in young patients. Orthopedics.

[69] Kayalar M., Ozerkan F., Bal E., Toros T., Ademoğlu Y., Ada S. (2008). Elbow arthrolysis in severely stiff elbows. Arch Orthop Trauma Surg.

[70] Rex C., Suresh Kumar P.M., Srimannarayana A., Chugh S., Ravichandran M., Harish D.N. (2008). Analysis of results of surgical treatment of posttraumatic stiff elbow. Indian J Orthop.

[71] Boerboom A.L., de Meyier H.E., Verburg A.D., Verhaar J.A. (1993). Arthrolysis for post-traumatic stiffness of the elbow. Int Orthop.

[72] Blauth M., Haas N.P., Südkamp N.P., Happe T. (1990). [Arthrolysis of the elbow in posttraumatic contracture]. Orthopä.

[73] Marti R.K., Kerkhoffs G.M., Maas M., Blankevoort L. (2002). Progressive surgical release of a posttraumatic stiff elbow. Technique and outcome after 2-18 years in 46 patients. Acta Orthop Scand.

[74] Phillips B.B., Strasburger S. (1998). Arthroscopic treatment of arthrofibrosis of the elbow joint. Arthroscopy.

[75] Tan V., Daluiski A., Simic P., Hotchkiss R.N. (2006). Outcome of open release for post-traumatic elbow stiffness. J Trauma.

[76] Sun Z., Cui H., Liang J., Li J., Wang X., Fan C. (2019). Determining the effective timing of an open arthrolysis for post-traumatic elbow stiffness: a retrospective cohort study. BMC Muscoskel Disord.

[77] Fan D., Wang W., Hildebrand K.A., Fan C.Y. (2016). Open arthrolysis for elbow stiffness increases carrying angle but has no impact on functional recovery. BMC Muscoskel Disord.

[78] Cai J., Zhou Y., Chen S. (2016). Ulnar neuritis after open elbow arthrolysis combined with ulnar nerve subcutaneous transposition for post-traumatic elbow stiffness: outcome and risk factors. J Shoulder Elbow Surg.

[79] Zheng W., Chen S., Song J., Liu J., Fan C. (2017). The influence of body mass index on outcome of open arthrolysis for post-traumatic elbow stiffness. J Shoulder Elbow Surg.

[80] Winkler S., Wagner F., Weber M. (2015). Current therapeutic strategies of heterotopic ossification--a survey amongst orthopaedic and trauma departments in Germany. BMC Muscoskel Disord.

[81] Joice M., Vasileiadis G.I., Amanatullah D.F. (2018). Non-steroidal anti-inflammatory drugs for heterotopic ossification prophylaxis after total hip arthroplasty: a systematic review and meta-analysis. Bone Joint Lett J.

[82] Zhu X.T., Chen L., Lin J.H. (2018). Selective COX-2 inhibitor versus non-selective COX-2 inhibitor for the prevention of heterotopic ossification after total hip arthroplasty: a meta-analysis. Medicine (Baltim).

[83] Rashid R.H., Qadir I., Ahmed W., Umer M. (2015). Prophylaxis against heterotopic ossification after elbow and acetabular fractures - do we really need it. J Pakistan Med Assoc.

[84] Cefo I., Eygendaal D. (2011). Arthroscopic arthrolysis for posttraumatic elbow stiffness. J Shoulder Elbow Surg.

[85] Amillo S. (1992). Arthrolysis in the relief of post-traumatic stiffness of the elbow. Int Orthop.

[86] Breitfuss H., Muhr G., Neumann K., Neumann C., Rehn J. (1991). [Arthrolysis of post-traumatic stiff elbow. Which factors influence the end result]. Unfallchirurg.

[87] Dávila S.A., Johnston-Jones K. (2006). Managing the stiff elbow: operative, nonoperative, and postoperative techniques. J Hand Ther.

[88] Everding N.G., Maschke S.D., Hoyen H.A., Evans P.J. (2013). Prevention and treatment of elbow stiffness: a 5-year update. J Hand Surg Am.

[89] Heirweg S., De Smet L. (2003). Operative treatment of elbow stiffness: evaluation and outcome. Acta Orthop Belg.

[90] Hertel R., Pisan M., Lambert S., Ballmer F. (1997). Operative management of the stiff elbow: sequential arthrolysis based on a transhumeral approach. J Shoulder Elbow Surg.

[91] Isa A.D., Athwal G.S., King G.J.W., MacDermid J.C., Faber K.J. (2018). Arthroscopic debridement for primary elbow osteoarthritis with and without capsulectomy: a comparative cohort study. Shoulder Elbow.

[92] Lindenhovius A.L., Doornberg J.N., Ring D., Jupiter J.B. (2010). Health status after open elbow contracture release. J Bone Joint Surg Am.

[93] Lindenhovius A.L., Jupiter J.B. (2007). The posttraumatic stiff elbow: a review of the literature. J Hand Surg Am.

[94] Park M.J., Kim H.G., Lee J.Y. (2004). Surgical treatment of post-traumatic stiffness of the elbow. J Bone Joint Surg Br.

[95] Schindler A., Yaffe B., Chetrit A., Modan M., Engel J. (1991). Factors influencing elbow arthrolysis. Ann Chir Main Memb Super.

[96] Ayadi D., Etienne P., Burny F., Schuind F. (2011). Results of open arthrolysis for elbow stiffness. A series of 22 cases. Acta Orthop Belg.

[97] Ruch D.S., Shen J., Chloros G.D., Krings E., Papadonikolakis A. (2008). Release of the medial collateral ligament to improve flexion in post-traumatic elbow stiffness. J Bone Joint Surg Br.

[98] Ring D., Hotchkiss R.N., Guss D., Jupiter J.B. (2005). Hinged elbow external fixation for severe elbow contracture. J Bone Joint Surg Am.

[99] Adams J.E., King G.J., Steinmann S.P., Cohen M.S. (2015). Elbow arthroscopy: indications, techniques, outcomes, and complications. Instr Course Lect.

[100] Gelinas J.J., Faber K.J., Patterson S.D., King G.J. (2000). The effectiveness of turnbuckle splinting for elbow contractures. J Bone Joint Surg Br.

[101] Ditsios K.T., Werner B.S., Yamaguchi K. (2011). [Arthroscopic capsular release of the elbow]. Orthopä.

[102] Kwak J.M., Kim H., Sun Y., Kholinne E., Koh K.H., Jeon I.H. (2020). Arthroscopic osteocapsular arthroplasty for advanced-stage primary osteoarthritis of the elbow using a computed tomography-based classification. J Shoulder Elbow Surg.

[103] Hertel R., Wolfram L. (2001). [Open arthrolysis in preserved joint surface geometry]. Orthopä.

[104] Gausepohl T., Mader K., Pennig D. (2006). Mechanical distraction for the treatment of posttraumatic stiffness of the elbow in children and adolescents. J Bone Joint Surg Am.

[105] Somanchi B.V., Funk L. (2008). Evaluation of functional outcome and patient satisfaction after arthroscopic elbow arthrolysis. Acta Orthop Belg.

[106] Kolb K., Koller H., Holz U. (2008). [Open arthrolysis of posttraumatic elbow stiffness]. Unfallchirurg.

[107] Nobuta S., Sato K., Kasama F., Hatori M., Itoi E. (2008). Open elbow arthrolysis for post-traumatic elbow contracture. Ups J Med Sci.

